# Facile storage and release of white phosphorus and yellow arsenic

**DOI:** 10.1038/s41467-017-02735-2

**Published:** 2018-01-24

**Authors:** Andreas E. Seitz, Felix Hippauf, Werner Kremer, Stefan Kaskel, Manfred Scheer

**Affiliations:** 1 Institute of Inorganic Chemistry, University of Regensburg, Universitätsstr. 31, 93040 Regensburg, Germany; 2 Faculty of Mathematics and Sciences, Discipline of Chemistry and Food Chemistry, Chair of Inorganic Chemistry I, Technical University of Dresden, Bergstr. 66, 01069 Dresden, Germany; 3 Institute of Biophysics and Physical Biochemistry, University of Regensburg, Universitätsstr. 31, 93040 Regensburg, Germany

## Abstract

The storage of metastable compounds and modifications of elements are of great interest for synthesis and other, e.g., semiconductor, applications. Whereas white phosphorus is a metastable modification that can be stored under certain conditions, storage of the extremely (light- and air-)sensitive form of arsenic, yellow arsenic, is a challenge rarely tackled so far. Herein, we report on the facile storage and release of these tetrahedral E_4_ molecules (E = P, As) using activated carbon as a porous storage material. These loaded materials are air- and light-stable and have been comprehensively characterized by solid-state ^31^P{^1^H} MAS NMR spectroscopy, powder X-ray diffraction analysis, nitrogen adsorption measurements, and thermogravimetric analysis. Additionally, we show that these materials can be used as a suitable E_4_ source for releasing intact white phosphorus or yellow arsenic, enabling subsequent reactions in solution. Because the uptake and release of E_4_ are reversible, these materials are excellent carriers of these highly reactive modifications.

## Abstract (web summary)

White phosphorus and yellow arsenic represent useful elemental sources for synthetic applications, but their poor stabilities make their storage highly challenging. Here, Scheer and colleagues encapsulate P_4_ and As_4_ molecules within porous activated carbons and demonstrate their use in subsequent chemical reactions.

## Introduction

Since the discovery of elemental white phosphorus^1^ (P_4_) by Hennig Brand in 1669, it has served as a widely used reagent in academia and industry alike. While activation of the P_4_ tetrahedron is of great interest in academic research^2–5^, industry is especially interested in the production of organophosphorus compounds and the synthesis of highly pure phosphoric acid starting from white phosphorus. However, solid P_4_ has to be handled with great care because of its high reactivity, its metastable character and its toxicity, and shipping even small amounts of P_4_ is subject to severe restrictions. P_4_ combusts spontaneously in air and decomposes slowly under light. As a result, P_4_ is stored under water in the dark. In 2009, Nitschke et al. showed the encapsulation of P_4_ within a self-assembled host–guest complex of iron, which is air-stable in the solid state^6^. The guest can be extracted by better fitting molecules such as benzene and cyclohexane, whereas poor fitting guests, such as *n*-heptane, cannot be used for extraction. Recently, Wu et al. succeeded in storing P_4_ and, interestingly, also As_4_ within tetrahedrally shaped cage complexes in solution^7, 8^. However, in all these cases, no subsequent reactions with the host–guest complexes were examined due to the resulting multi-component mixtures of the overall systems (occurrence of side reactions). Moreover, the Kawano group showed the encapsulation of P_4_ in the reusable metal–organic framework (MOF) [(ZnI_2_)_3_(TPT)]_n_ (TPT = 2,4,6-tris(4-pyridiyl)-1,3,5-triazine)^9^, again without using them for subsequent syntheses. Here, P_4_ was synthesized from red phosphorus and trapped by gas phase diffusion. Furthermore, we succeeded in the encapsulation and stabilization of intact tetrahedral E_4_ molecules (E = P, As) in polymeric and spherical aggregates constructed by copper halides and [Cp*Fe(η^5^-P_5_)] (Cp* = η^5^-C_5_Me_5_)^10^. Even if the corresponding crystals are air and light-stable, they are insoluble in common solvents and the group 15 tetrahedra are not releasable without the decomposition of the complexes. Moreover, together with other coinage metal P_4_ complexes, the air-sensitive silver(I) salts [Ag(η^2^-E_4_)_2_][pftb] (E = P, As; pftb = [Al{OC(CF_3_)_3_}_4_]) show to be suitable sources of intact E_4_ (E = P, As) for particular chemical reactions, however with a limited scope due to the need of initial AgCl elimination^11–14^. Recently, we found two other systems, [(LCu)_2_(μ,η^2:2^-E_4_)] (E = P, As; *L* = [{N(C_6_H_3_*i*Pr_2_-2,6)C(Me)}_2_CH^−^]) and Cp^R^E_4_ (E = P; Cp^R^ = Cp* (η^5^-C_5_Me_5_), Cp″′ (η^5^-C_5_H_3_*t*Bu_2_), Cp^4*i*Pr^ (η^5^-C_5_H*i*Pr_4_), Cp^BIG^ (η^5^-C_5_(4-*n*BuC_6_H_4_)_5_); E = As, Cp^R^ = Cp^PEt^ (η^5^-C_5_(4-EtC_6_H_4_)_5_)), which coordinate either side-on the intact E_4_ tetrahedra or radically opened an E–E bond to form a twofold organic-substituted compound with a tetraphospha/tetraarsabicyclo[1.1.0]butane as a central structural motif^15–17^. For both systems, we could show the release of intact E_4_ tetrahedra, irreversible in solution, but reversible in the solid state for the latter case (except for E = As). However, all known coordinating/encapsulating systems of intact E_4_ have the disadvantage of a multistep synthesis of the starting materials before the final E_4_ addition can be executed, which is preparatively time-consuming, expensive, and surely not feasible in large-scale batches. Moreover, the existence of additional reactive components in the solutions after the release of E_4_ makes these systems unusable for subsequent reactions.

In contrast to white phosphorus, the heavier congener As_4_ has less been used in industry and academia so far because of its pronounced photosensitivity and its extremely difficult and inconvenient handling. Yellow arsenic, discovered as an element modification 150 years ago^18^, is a metastable compound rapidly decomposing in the solid state even in the dark at low temperatures. Additionally, exposed to light, it decomposes within minutes in solution at room temperature into the thermodynamically stable modification gray arsenic^19^. Whereas solid yellow arsenic can practically not be stored, only solutions of freshly prepared As_4_ can be used for subsequent reactions^20^, which can, however, only be performed in the absolute dark without any exposure to light whatsoever, and the used amount of As_4_ is not exactly quantifiable, due to its fast decomposition to gray arsenic. Therefore, the challenging quest arises to develop an easily accessible and light-stable storage and release material for yellow arsenic to open ample perspectives for the usage of this very reactive modification in syntheses and reactivity by a large chemistry community. Keeping in mind the extremely low solubility of As_4_ at low temperatures, the need for a usable As_4_ delivery source to overcome this difficulty is obvious. Moreover, for both modifications, the problem of a possible hazard-free shipping has to be solved, which would be a breakthrough regarding their application by both industry and academia.

Herein, we report on the facile storage and release of yellow arsenic and white phosphorus, respectively, using activated carbon as a porous storage material, and on the first use of these materials for subsequent reactions in solutions.

## Results

### Preparation

The loaded materials E_4_@**C** (E = P, As) can be easily prepared by adsorbing E_4_ (E = P, As) from a freshly prepared solution in tetrahydrofuran (THF) by an activated carbon material (**C**) with a defined pore size distribution resulting in an air- and light-stable black solid of E_4_@**C** after work-up (Supplementary Figs. 1 and 2).

### Release of E_4_

To study the release of E_4_ molecules by sublimation, the E_4_@**C** (for E = P: 820 mg; As: 418 mg) material was sublimed in vacuo at oil bath temperature of 160 °C and cold finger temperature of −20 °C. After 2 days, a black solid remained in the sublimation apparatus (for E = P: 508 mg; E = As: 370 mg), whereas 300 mg of a white waxy solid or 28 mg of a gray amorphous solid was isolated from the cold finger for E = P or As, respectively. The white waxy solid was characterized by ^31^P NMR spectroscopy well corresponding to those of P_4_ (Supplementary Fig. 3). The gray solid was characterized by powder X-ray diffraction analysis proving that it is amorphous arsenic (Supplementary Figs. 4 and 5). Furthermore, the solid was identified as gray arsenic by ICP-OES (inductively coupled plasma-optical emission spectrometry, Supplementary Fig. 6).

As the P_4_@**C** material retained molecular P_4_, a standard procedure was used to extract it with common solvents as *n*-hexane, toluene, dichloromethane, and THF. For this reason, P_4_@**C** (100 mg) was dispersed in exactly 10 mL of each of these solvents and stirred for 24 h in the dark. A ^31^P NMR spectrum of a defined volume (the same in all cases) of the supernatant was recorded using the same standard C_6_D_6_ capillary with defined internal PPh_3_. In the case of CS_2_, 152 mg P_4_@**C** in 20 mL solvent were used (Supplementary Figs. 7–11).

### Stability

The stability of P_4_@**C** was examined by storing some black solid on the bench in air and under light for 3 weeks. This sample neither ignites spontaneously when brought to air nor reacts with a tissue over time (Supplementary Fig. 17). The solid was then examined with ^31^P{^1^H} MAS NMR. A signal for P_4_ is only slightly (by 8 ppm) down-field shifted compared to the inert stored sample. This fact can be attributed to a slightly different interaction in air within the pores (Supplementary Fig. 18). Additionally, solely one sharp signal for a decomposition product at 1.5 ppm is detected. Presumably, it can be attributed to H_3_PO_4_ immobilized within the carbon scaffold, since the signal is in the known range for phosphate derivatives^21^. This may be a result of slow oxidation and hydrolysis to some extent on the surface of the scaffold bound P_4_. However, it has to be noted that a signal for white phosphorus can be still detected after storage for 3 weeks in air. Furthermore, P_4_@**C** (88 mg) was brought again under nitrogen atmosphere and slurried in 7 mL CS_2_ overnight followed by ^31^P NMR investigation of the colorless supernatant. The same standard C_6_D_6_ capillary with defined internal PPh_3_ content was used (Supplementary Fig. 19). An intense signal characteristic of free P_4_ was again monitored. These results clearly prove the stability of P_4_@**C** in light and in air to some extent for at least 3 weeks. However, storage in air is not recommended due to a possible decomposition over a long period of time.

In the case of arsenic, no spontaneous ignition of As_4_@**C** was observed. The material was exposed to light for several days and stored in air, though in a fume hood due to its unknown toxicity. However, after extraction with CS_2_, no signal for As_4_ was monitored by ^75^As NMR spectroscopy. Consequently, the extracted As_4_ is likely harmed by slight oxygen and to some extent moisture uptake within the scaffold. Nevertheless, after sublimation for 48 h at the above-mentioned conditions, resulting gray solid being undoubtedly authenticated by ICP-OES as gray arsenic (Supplementary Figs. 20 and 21).

### Recycling

The used storage material **C** can be recycled and used again for storage purposes, especially for phosphorus. After washing E_4_ out of E_4_@**C**, the regained storage material **C** is to be heated for several hours in high vacuum before it can be again loaded with solutions of E_4_.

## Discussion

**Fig. 1:**
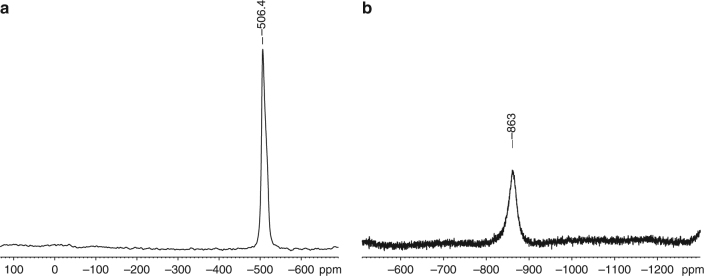
NMR spectroscopy. **a** ^31^P{^1^H} MAS NMR spectrum of P_4_@**C**. **b** ^75^As NMR spectrum of As_4_ in CS_2_ extracted from As_4_@**C**

For P_4_@**C**, the decrease of the P_4_ concentration of the THF supernatant during the synthesis of the loaded material was determined by ^31^P NMR spectroscopy, indicating a minimum uptake of at least 18% phosphorus by mass. This result is in accordance with the elemental analysis of P_4_@**C** indicating ~20% phosphorus by mass, which is considerably more than for the tetranuclear iron complex described, e.g., by Nitschke with only 3.5% phosphorus by mass or for the P_4_-MOF complex by Kawano with 6.9% phosphorus by mass^6, 9^. The solid state ^31^P{^1^H} MAS NMR spectrum of P_4_@**C** reveals solely a broad singlet with a chemical shift of −506.4 ppm (*ω*_1/2_ = 1582 Hz) (Fig. 1a), which is in good agreement with the reported chemical shift of the encapsulated P_4_ molecule in spherical aggregates (−506 ppm)^10^. The signal is high-field shifted compared to P_4_ both as a solid (−462 ppm) and a liquid (−460 ppm), due to the interaction with its environment^22–25^. In contrast, the signal is low-field shifted compared to P_4_ in the gas phase (−553 ppm) and in solution (e.g., in benzene −522 ppm)^23, 26^. For the arsenic compound As_4_@**C**, no assignable signal can be detected in the solid-state ^75^As MAS NMR spectrum, which can be attributed to the low sensitivity of the quadrupolar nucleus ^75^As (*Q*_spec_ = 0.314(6) *b* = 0.314(6) × 10^−28^ m^2^)^27^. However, a broad signal for free As_4_ can be detected in the ^75^As NMR spectrum at −863 ppm (*ω*_1/2_ = 2060 Hz) by extraction of As_4_@**C** with CS_2_ (Fig. 1b). Interestingly, this NMR spectrum was recorded after the storage of the As_4_ containing material in a glove box in the presence of light for more than 2 weeks. Consequently, As_4_@**C** can be seen as light-stable storage material of intact yellow arsenic. The chemical shift is also in good agreement with the reported values (−892 ppm for toluene/CD_2_Cl_2_ and −908 ppm for THF/CD_2_Cl_2_, respectively) considering the influence of the different solvents^10^.

**Fig. 2:**
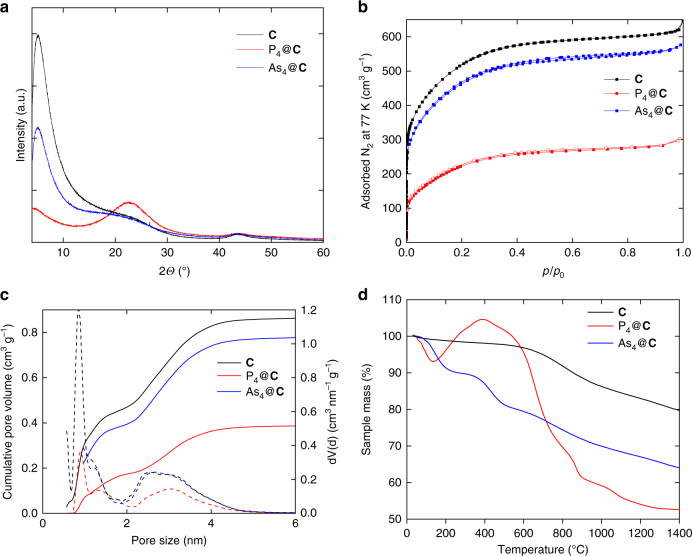
Comparisons of unloaded carbon material **C** (black), P_4_@**C** (red) and As_4_@**C** (blue). **a** Powder X-ray diffractograms. **b** Nitrogen physisorption isotherms at 77 K. **c** DFT pore size distribution. **d** Thermogravimetric curve

The powder X-ray diffractograms of E_4_@**C** (E = P, As) show only two broad peaks arising from the amorphous carbon material and no diffraction pattern of E_4_ units (Fig. 2a). Thus, no crystalline E_4_ is present in the scaffold, indicating that the arsenic and phosphorus tetrahedra, respectively, are located in the pores (<5 nm). To clarify the distribution of the E_4_ (E = P, As) molecules, the nitrogen adsorption measurement at liquid nitrogen temperature of E_4_@**C** was examined and the DFT (density functional theory)-based pore size distribution was determined (Fig. 2b). As a result of the P_4_ storage, the specific surface area and the total pore volume of the carbon host decrease by 55% and 57%, respectively. Considering the bulk density of P_4_ (1.82 g cm^−3^)^28^, the maximum amount of P_4_ in P_4_@**C** is approximately 36%. The DFT pore size distribution displays the lack of the ultramicropores (<0.7 nm) as well as an intense decrease of supermicropores (0.7 < 2 nm) (Fig. 2c). Considering a P-P bond length of approx. 2.21 Å and a van der Waals radius of 1.80 Å for a phosphorus atom, the size of the P_4_ molecules (tetrahedrally shaped sphere with a diameter of about 0.61 nm) is similar to the pore size of the carbon host enabling a good fit into the ultramicropores^29, 30^. Additionally, the amount of meso pores (>2 nm) diminishes. These results prove the incorporation of the P_4_ molecules into the porous activated carbon. For As_4_@**C**, the specific surface area and the total pore volume decrease by 9% and 14%, respectively. Considering the bulk density of As_4_ (1.97 g cm^−3^)^28^, the maximum amount of As_4_ in As_4_@**C** is approximately 10%. With a diameter of about 0.65 nm, As_4_ molecules smoothly fit into the carbon scaffold. The thermogravimetric analysis of As_4_@**C** shows two steps of a loss of weight. In a first step, until 350 °C, the sample loses approximately 10% of its weight followed by an additional loss of 10% until 600 °C (Fig. 2d). In contrast, the thermogravimetric analysis of solid P_4_@**C** shows a loss of weight of about 10% until 180 °C followed by an unexpected, but reproducible increase of the sample mass by 10% (Fig. 2d). Between 400 and 800 °C, a loss of weight of 32% is again detected resulting from released phosphorus.

Additionally, the suitability of E_4_@**C** for the preparative release of intact white phosphorus and yellow arsenic was investigated. By sublimation, a white waxy solid of P_4_ can be isolated within two days at 160 °C in vacuum. The ^31^P NMR spectrum of this solid, dissolved in benzene-*d*_6_ (singlet at −519.5 ppm), proves unambiguously that the P_4_ tetrahedra are still intact. The so obtained amount of phosphorus (36% by mass) indicates a maximum amount for the uptake of the porous carbon material and is in very good agreement with the calculated value of incorporated white phosphorus. The analogous sublimation with As_4_@**C** leads to the release of As_4_ and the isolation of at least 7% arsenic by mass. Due to the instability of yellow arsenic under light exposure, only a gray amorphous solid was isolated, which can be attributed to gray arsenic by ICP-OES. Furthermore, the extraction of P_4_ by different common solvents such as CS_2_, *n*-hexane, toluene, dichloromethane and THF was explored. A standard procedure for these solvents reveals that 17–21% P_4_ by mass can be extracted. In the case of yellow arsenic, extraction with common solvents as for instance *n*-pentane and toluene showed experimentally a release of about 5–10% arsenic by mass.

**Fig. 3:**
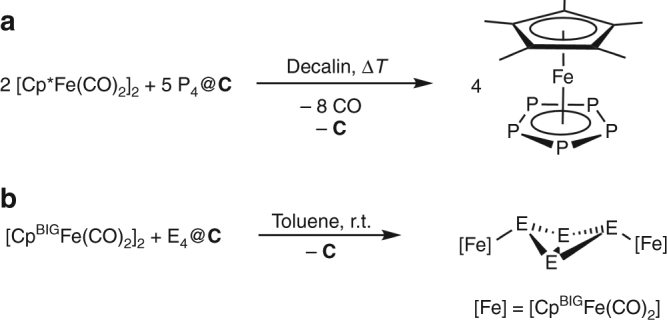
Reactivity studies. **a** Synthesis of [Cp*Fe(η^5^-P_5_)]. **b** Synthesis of [(Cp^BIG^Fe(CO)_2_)_2_(µ,η^1:1^-E_4_)] (E = P, As)

Additionally, we were interested in the use of E_4_@**C** for subsequent chemical reactions because activated carbon as an inert material should not harm chemical reactions by unintended contaminations in the way different metal ions do. Compared to known storage materials, which are based on multi-component systems containing metal ions, our system solely consists of an inert carbon host thus not resulting in any competitive reactions. That way, we succeeded in the synthesis of the reported compounds starting from E_4_@**C** (E = P, As). For example, the reaction of P_4_@**C** with [Cp*Fe(CO)_2_]_2_ in boiling decalin leads to the formation of the desired pentamethylcyclopentadienyl-pentaphosphaferrocene [Cp*Fe(η^5^-P_5_)] (Fig. 3a)^31^. Additionally, P_4_@**C** was reacted with [Cp^BIG^Fe(CO)_2_]_2_ (Cp^BIG^ = C_5_(4-*n*BuC_6_H_4_)_5_) at room temperature, analogously to the reported procedure^32^ starting from dissolved P_4_, resulting in the quantitative synthesis of the P_4_ butterfly complex [(Cp^BIG^Fe(CO)_2_)_2_(μ,η^1:1^-P_4_)] (Fig. 3b). The analogous reaction was also observed for As_4_@**C**. Therefore, E_4_@**C** has been shown to be a convenient source of white phosphorus and yellow arsenic for subsequent reactions. Because As_4_@**C** can also be used to deliver yellow arsenic at low temperatures for chemical syntheses, it becomes a useful synthetic tool and opens broad perspectives for its application.

Moreover, P_4_@**C** can be stored for at least four weeks in light and in air, without the black solid igniting spontaneously in air. After the storage of P_4_@**C** for three weeks in air on a bench, the solid-state ^31^P{^1^H} MAS NMR spectrum still reveals one broad singlet with a chemical shift of −498.4 ppm, which can be attributed to white phosphorus P_4_. This signal is only slightly shifted compared to the signal of P_4_@**C** stored in a glove box and is still in good agreement with the reported chemical shifts of P_4_. However, solely one additional very weak signal at 1.5 ppm was detected resulting presumably from traces of the oxidation of solid P_4_ and subsequent hydrolysis to some extent. Nevertheless, P_4_ can be still extracted with CS_2_ monitored by the ^31^P NMR spectroscopy and no decomposition product of P_4_ was observed by the solution ^31^P NMR spectroscopy in contrast to the solid-state NMR spectroscopy. Principally, As_4_@**C** can also be stored in light and in air, but the storage in air is not recommended due to its unknown toxicity. The stability of the E_4_ tetrahedra in the porous material might be attributed to its interaction within the pores of the carbon material since the tetrahedral structure and size of the E_4_ molecules match nearly perfectly the size of the pores and the cavities of the carbon scaffold, respectively. Anyhow, long-time storage of E_4_@**C** in air should be avoided due to a possible slow decomposition process.

In summary, we have shown the facile storage and release of yellow arsenic and white phosphorus in an inert and low-cost activated carbon-based porous material. Because of the stabilization of these metastable compounds in a porous framework, we succeeded in synthesizing a light-stable, storable and conveyable P_4_ and As_4_ source. This allows for example unproblematic shipping, especially of white phosphorus, which is hindered by law for small batches, and renders possible the shipping of yellow arsenic as a stabilized material. Additionally, these sources can be used directly for chemical reactions. As a result, new applications for phosphorus and arsenic chemistry are opened up for academia and industry. That way, the As_4_ containing material is especially regarded as a suitable arsenic source with a high potential, e.g., for new approaches in arsenic chemistry and, for instance, in semiconductor technologies e.g., for chemical vapor deposition (CVD) processes. Because of the multiple use of the carbon-based material for storage and release, it becomes an extremely useful and low-cost solid for these purposes. The uploading of other unstable molecules might create important perspectives thus representing a topic of future investigations.

## Methods

### General

All manipulations were performed under an atmosphere of dry nitrogen and under exclusion of oxygen and moisture using standard Schlenk and glove box techniques. All solvents were dried using conventional techniques, degassed and saturated with nitrogen prior to use.

### Activation of porous carbon material

The porous carbon material (**C**, commercially available Norit DLC Super 50 of the Cabot Norit Nederland BV) was activated in vacuo at 140 °C before utilization.

### Characterization

NMR spectroscopy: ^1^H, ^31^P{^1^H} and ^31^P NMR spectra were recorded on a Bruker Avance 400 (^1^H: 400.130 MHz, ^31^P: 161.976 MHz) or on a Bruker Avance 300 (^1^H: 300.132 MHz, ^31^P: 121.495 MHz) spectrometer. The chemical shifts are reported in ppm relative to external TMS (^1^H) and 85% H_3_PO_4_ (^31^P). The ^75^As NMR spectra were recorded on a Bruker Avance III HD 600 (600.13 MHz) spectrometer, equipped with a 5 mm TBI-probehead (^1^H, X, ^19^F) and (^1^H, X, ^31^P) with Z-gradient, respectively, and externally referenced to Na[AsF_6_]. The ^31^P{^1^H} MAS NMR spectra were recorded on a Bruker Avance 300 MHz device and referenced to external NaH_2_PO_4_ (2.3 ppm).

IR spectroscopy: The IR spectra were measured on a VARIAN FTS-800 FT-IR spectrometer.

Raman spectroscopy: The Raman spectra were recorded on a Thermo Fisher Scientific DXR-Smart-Raman spectrometer (excitation laser 532 nm; Supplementary Fig. 22).

EPR spectroscopy: The X-Band EPS spectra were recorded on a Magnettech MiniScope MS400 spectrometer with 9 GHz microwave frequency (Supplementary Fig. 23).

Elemental analysis: The elemental analysis was performed by the Catalysis Research Center of the Technical University of Munich. Phosphorus was thereby photometrically determined by the P-vanadate method. C, H and N were determined by combustion analysis. The values are seen as benchmarks since the sample was not totally stable during weighing.

Powder X-ray diffraction analysis: Powder X-ray data were collected using a XʹPert Pro diffractometer from PANalytical with Cu_Kα1_ radiation (*λ* = 1.5406 Å) in reflection mode from 4° to 60° 2*θ*.

Physisorption and DFT pore size distribution: Prior to porosity analysis, porous samples were degassed under vacuum at room temperature for 24 h. Nitrogen physisorption measurements at −196 °C were carried out on a Quadrasorb apparatus (Quantachrome Instruments) and an Autosorb 1 C (Quantachrome Instruments) for low-pressure experiments. Specific surface areas were calculated using the micropore BET assistant of the ASiQwin 3.01 software from Quantachrome to find the optimum *P*/*P*_0_. Total pore volumes were determined from the amount of adsorbed nitrogen at 0.95 *P*/*P*_0_. Pore size distributions (PSDs) were calculated using the quenched solid density functional theory method for carbon (slit/cylindrical pores, adsorption branch). Micropore volumes are estimated from the cumulative pore volumes at a diameter of 2 nm.

Thermogravimetric analysis: TG-DTA under argon atmosphere was measured with a Netzsch STA 409 PC/PG. An empty corundum crucible was used as a reference and the heating ramp was 1 K min^−1^.

### Syntheses

Synthesis of P_4_@**C**: A solution of P_4_ (595 mg, 4.80 mmol) in 40 mL THF was added to activated **C** (975 mg) and stirred for 17 h in the dark. Afterward, the black slurry was centrifuged and the supernatant was decanted. The resulting black solid was washed with *n*-pentane (2 × 10 mL) and dried in vacuo. Yield: 1.46 g. For the determination of the decrease of white phosphorus, ^31^P NMR spectra of the supernatant before and after addition of the solution to the activated carbon were recorded (Supplementary Figs. 1 and 2). Hereby, defined sample volumes were used and a standard C_6_D_6_ capillary with defined amount of PPh_3_ as internal standard was utilized. ^31^P{^1^H} MAS NMR (121 MHz, 300 K): *δ* [ppm] = −506.4 (s, br, *ω*_1/2_ = 1582 Hz, P_4_); elemental analysis (%): found: C, 72.66; H, 0.93; N, 0.18; P, 20.45.

Synthesis of yellow arsenic: Solutions of yellow arsenic were prepared according to literature procedures^19, 33^.

Synthesis of As_4_*@***C**: A freshly prepared solution of As_4_ (ca. 150 mg, starting from ~6–8 g gray arsenic) in 250 mL THF was filtered over diatomaceous earth and added to activated **C** (1.0 g). The black suspension was then stirred for ~17 h in the dark. Subsequently, the slurry was centrifuged and the supernatant was decanted. Afterward, the black solid was washed with *n*-pentane (1 × 30 mL) and dried in vacuo. Yield: 1.1 g.

Synthesis of [Cp*Fe(η^5^-P_5_)]: P_4_@**C** (661 mg) and [Cp*Fe(CO)_2_]_2_ (50 mg, 0.10 mmol) were refluxed in boiling decalin (30–40 mL) for 2 h. Subsequently, the solvent was removed resulting in a gray black solid. The latter was slurried in dichloromethane and filtered over diatomaceous earth and then over SiO_2_. The green filtrate was brought to dryness resulting in a dark green solid. The NMR chemical shifts (NMR spectra: Supplementary Figs. 12 and 13) are in very good agreement with the literature values^31^. Yield: 5 mg (0.014 mmol, 7%). ^1^H NMR (CD_2_Cl_2_, 400 MHz, 300 K): *δ* [ppm] = 1.43 (s, 15 H, C_5_(CH_3_)_5_); ^31^P{^1^H} NMR (CD_2_Cl_2_, 161 MHz, 300 K): *δ* [ppm] = 152.2 (s, *P*_5_); ^31^P NMR (CD_2_Cl_2_, 161 MHz, 300 K): *δ* [ppm] = 152.2 (s, *P*_5_).

Synthesis of [{Cp^BIG^Fe(CO)_2_}_2_(μ,η^1:1^-P_4_)]: A green solution of [Cp^BIG^Fe(CO)_2_]_2_ (Cp^BIG^ = C_5_(4-*n*BuC_6_H_4_)_5_) (100 mg, 0.06 mmol) in toluene (10 mL) was added to solid P_4_@**C** (40 mg). Immediately, the supernatant of the black suspension turned orange. The suspension was brought to dryness and slurried in dichloromethane. Then, the slurry was filtered over diatomaceous earth. The orange filtrate was brought to dryness. The analytical data are in very good agreement with the reported values (NMR spectra: Supplementary Figs. 14 and 15)^32^. Yield: 98 mg (0.054 mmol, 91% based on [Cp^BIG^Fe(CO)_2_]_2_). ^1^H NMR (C_6_D_6_, 400 MHz, 300 K): *δ* [ppm] = 0.80 (t, ^3^*J*(H,H) = 7.3 Hz, 30 H, CH_3_), 1.16 (m, 20 H, CH_2_), 1.35 (m, 20 H, CH_2_), 2.29 (t, ^3^*J*(H,H) = 7.8 Hz, 20 H, CH_2_), 6.72 (d, ^3^*J*(H,H) = 8.0 Hz, 20 H, C_6_H_4_), 7.32 (d, ^3^*J*(H,H) = 8.0 Hz, 20 H, C_6_H_4_); ^31^P NMR (C_6_D_6_, 161 MHz, 300 K): *δ* [ppm] = −54.1 (t, ^1^*J*(P,P) = 187 Hz, 2 P, Fe–*P*–P), −317.2 (t, ^1^*J*(P,P) = 187 Hz, 2 P, Fe–P–*P*); ^31^P{^1^H} NMR (C_6_D_6_, 161 MHz, 300 K): *δ* [ppm] = −54.1 (t, ^1^*J*(P,P) = 187 Hz, 2 P, Fe–*P*–P), −317.2 (t, ^1^*J*(P,P) = 187 Hz, 2 P, Fe–P–*P*); IR (toluene): $\tilde v$ [cm^−1^] = 2002 (s), 1955 (s).

Synthesis of [{Cp^BIG^Fe(CO)_2_}_2_(μ,η^1:1^-As_4_)]: A green solution of [Cp^BIG^Fe(CO)_2_]_2_ (Cp^BIG^ = η^5^-C_5_(4-*n*BuC_6_H_4_)_5_) (100 mg, 0.06 mmol) in toluene (10 mL) was added to solid As_4_@**C** (400 mg). Thereby, the supernatant of the black suspension turned red-orange. The suspension was brought to dryness and slurried in dichloromethane. Then, the slurry was filtered over diatomaceous earth. The red-orange filtrate was brought to dryness. The analytical data are in very good agreement with the reported values (NMR spectra: Supplementary Fig. 16)^32^. Yield: 93 mg (0.047 mmol, 78% based on [Cp^BIG^Fe(CO)_2_]_2_). ^1^H NMR (C_6_D_6_, 400 MHz, 300 K): *δ* [ppm] = 0.80 (t, ^3^*J*(H,H) = 7.2 Hz, 30 H, CH_3_), 1.15 (m, 20 H, CH_2_), 1.33 (m, 20 H, CH_2_), 2.27 (t, ^3^*J*(H,H) = 7.8 Hz, 20 H, CH_2_), 6.71 (d, ^3^*J*(H,H) = 8.0 Hz, 20 H, C_6_H_4_), 7.32 (d, ^3^*J*(H,H) = 8.0 Hz, 20 H, C_6_H_4_); IR (toluene): $\tilde v$ [cm^−1^] = 1993 (s), 1947 (s).

### Data availability

The data that support the findings of this study are available from the corresponding author on reasonable request; see author contributions for specific data sets.

## Electronic supplementary material

Supplementary Information
