# Supplementary material for: Facile storage and release of white phosphorus and yellow arsenic
Source: Nat Commun. 2018 Jan 24;9:361. doi: 10.1038/s41467-017-02735-2 (PMC5783940; doi:10.1038/s41467-017-02735-2)
Supplement: Supplementary file 1 — Supplementary Information [file 41467_2017_2735_MOESM1_ESM.pdf]

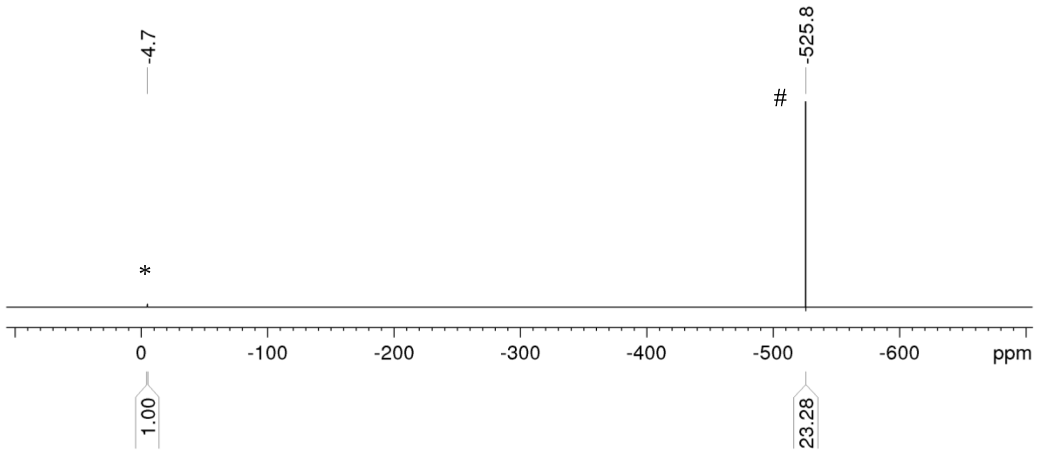


**Supplementary Figure 1.** $^{31}P$ NMR spectra of the $P_4$ (#) containing supernatant solution before addition to activated solid **C.** The NMR spectrum was recorded in thf with a $C_6D_6$ capillary including $PPh_3$ (*) as internal standard.

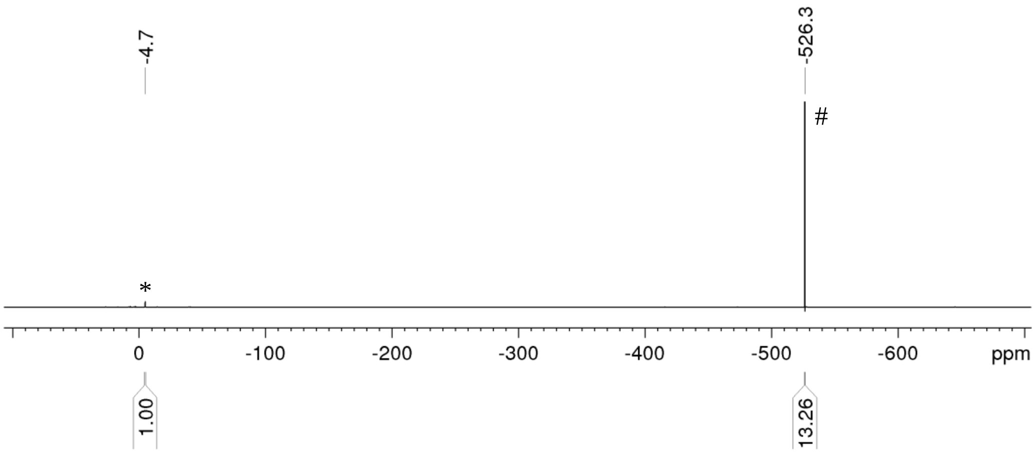


**Supplementary Figure 2.** $^{31}P$ NMR spectra of the $P_4$ (#) containing supernatant solution after addition to activated solid **C.** The NMR spectrum was recorded in thf with a $C_6D_6$ capillary including $PPh_3$ (*) as internal standard.

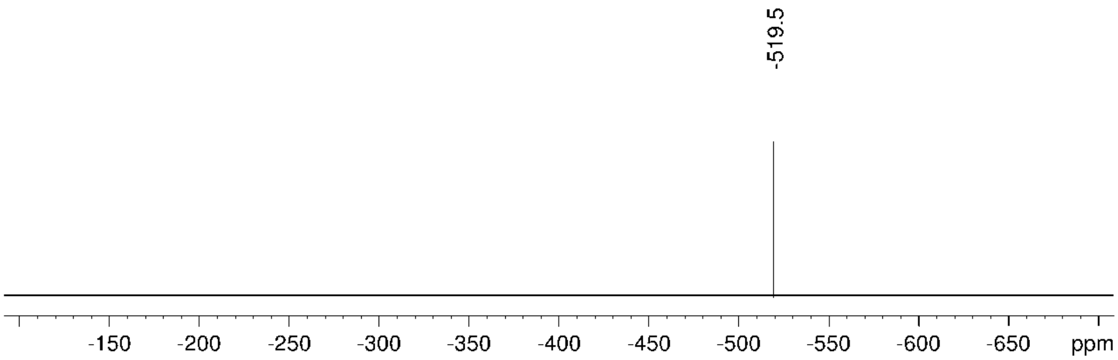


**Supplementary Figure 3.** $^{31}P$ NMR spectrum of the white waxy solid in $C_6D_6$ at 300 K. $^{31}P$ NMR ($C_6D_6$, 161 MHz, 300 K): $\delta$ [ppm] = -519.5 (s, $P_4$).

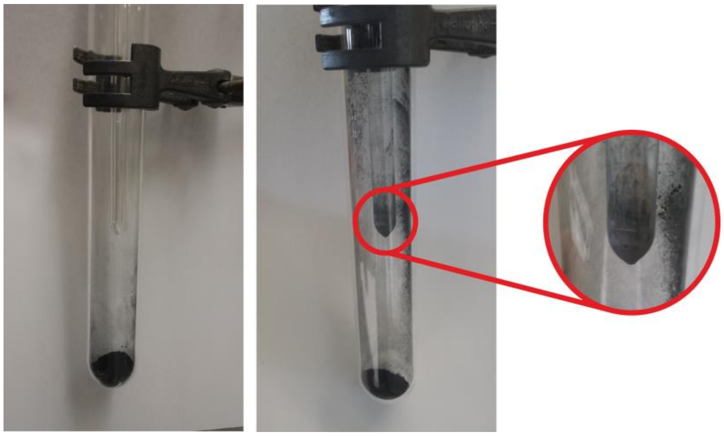

**Supplementary Figure 4.** Pictures of the sublimation apparatus before (left) and after (right) sublimation of $As_4$@**C**. The red circle highlights the grey solid which covers the cold finger after sublimation.

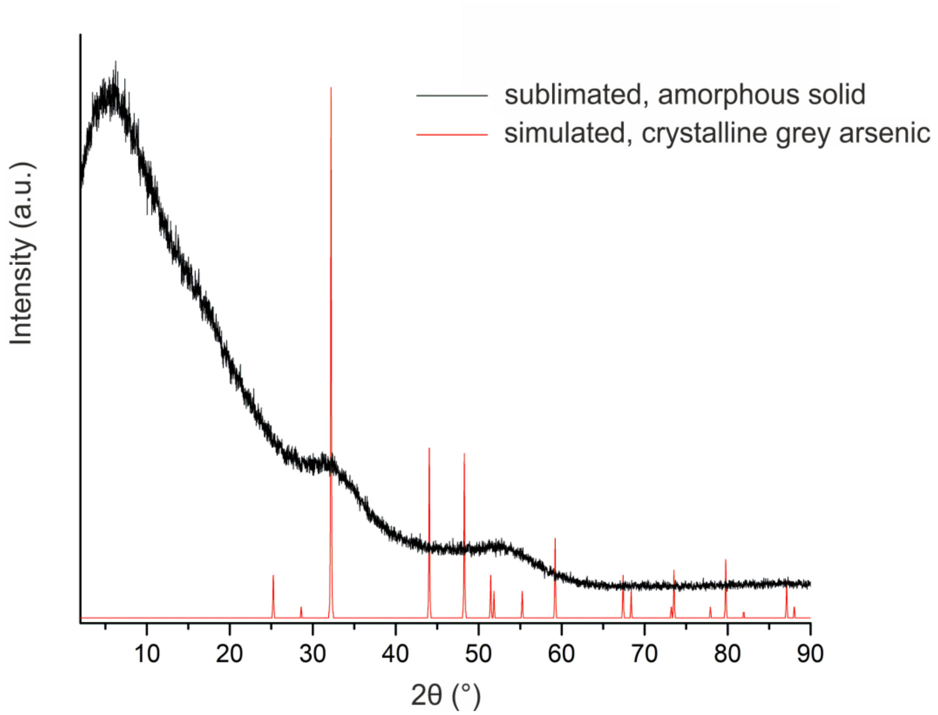


**Supplementary Figure 5.** Powder X-ray diffractogram of the amorphous grey solid (up) and simulated diffractogram of crystalline grey As (down).

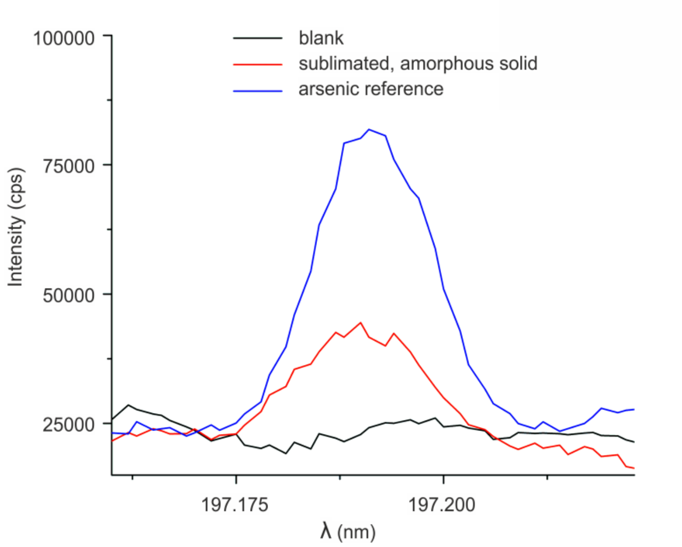


**Supplementary Figure 6.** Spectrum of the ICP-OES measurement of the blank (black), the dissolved sublimated amorphous solid (red) and the arsenic reference (blue). Therefore, the sublimated solid was dissolved in concentrated $HNO_3$ and the solution was measured on a Spectro-Flame EOP. Additionally, pure $HNO_3$ (blank) and a 100 µM solution of $As_2O_3$ in concentrated $HNO_3$ (arsenic reference) were measured. The characteristic emission of arsenic at $\lambda$ = 197.197 nm was investigated.

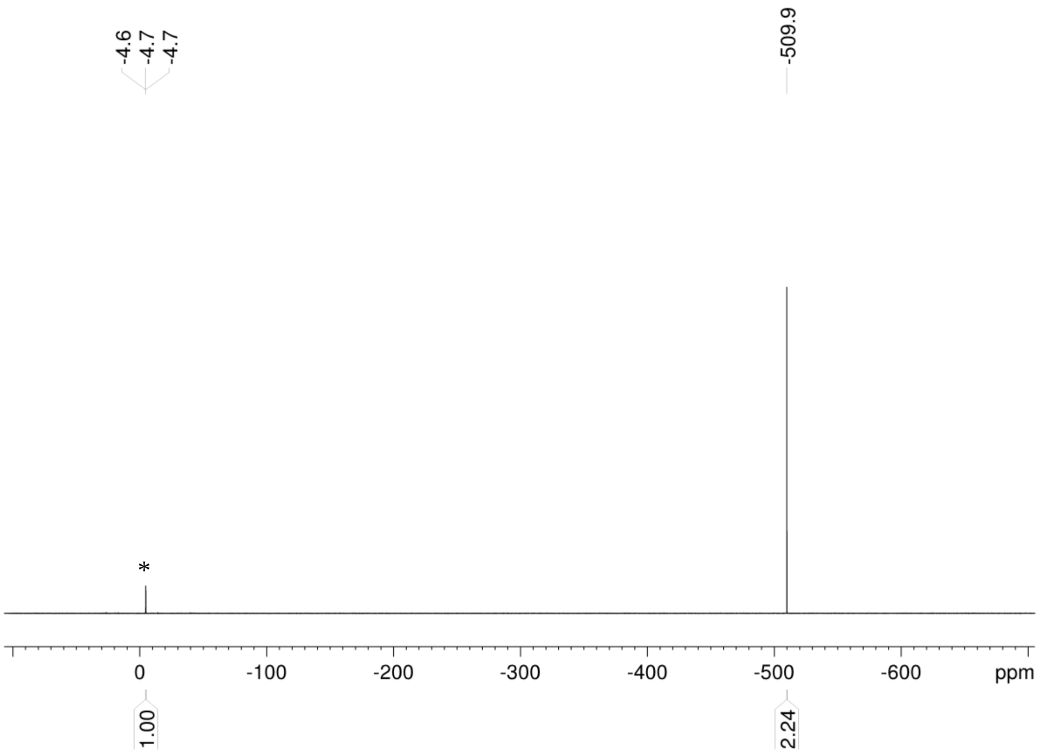


**Supplementary Figure 7.** $^{31}P$ NMR spectrum after extraction of $P_4$@**C** with $CS_2$. The NMR spectrum was recorded with a $C_6D_6$ capillary including $PPh_3$ (*) as internal standard.

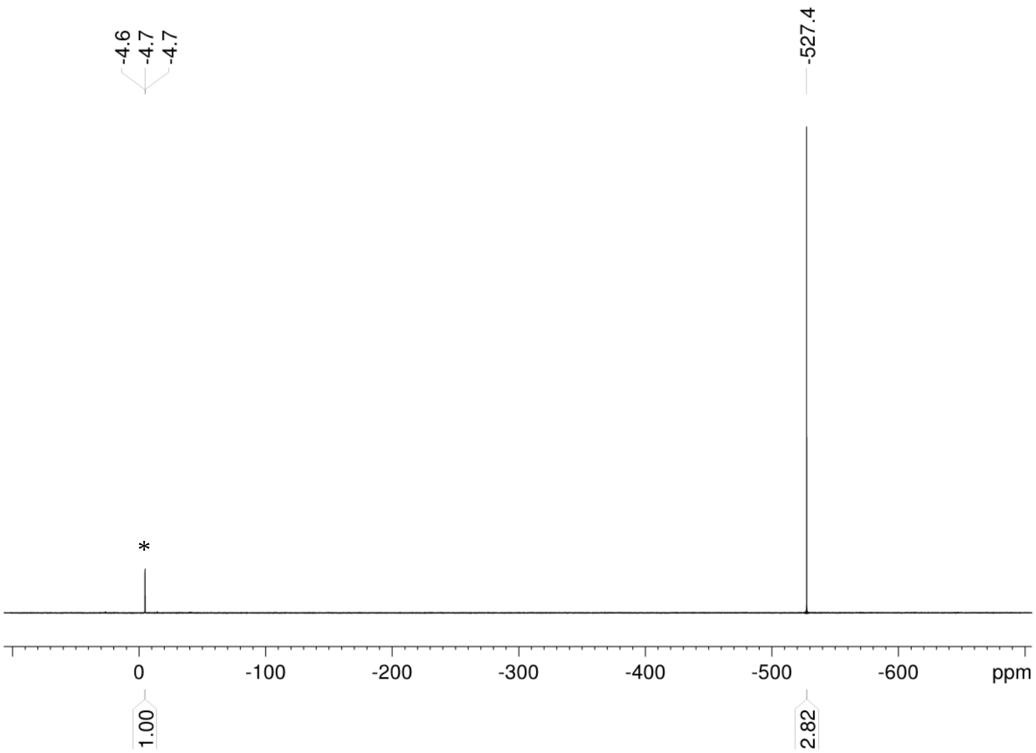


**Supplementary Figure 8.** $^{31}P$ NMR spectrum after extraction of $P_4$@**C** with *n*-hexane. The NMR spectrum was recorded with a $C_6D_6$ capillary including $PPh_3$ (*) as internal standard.

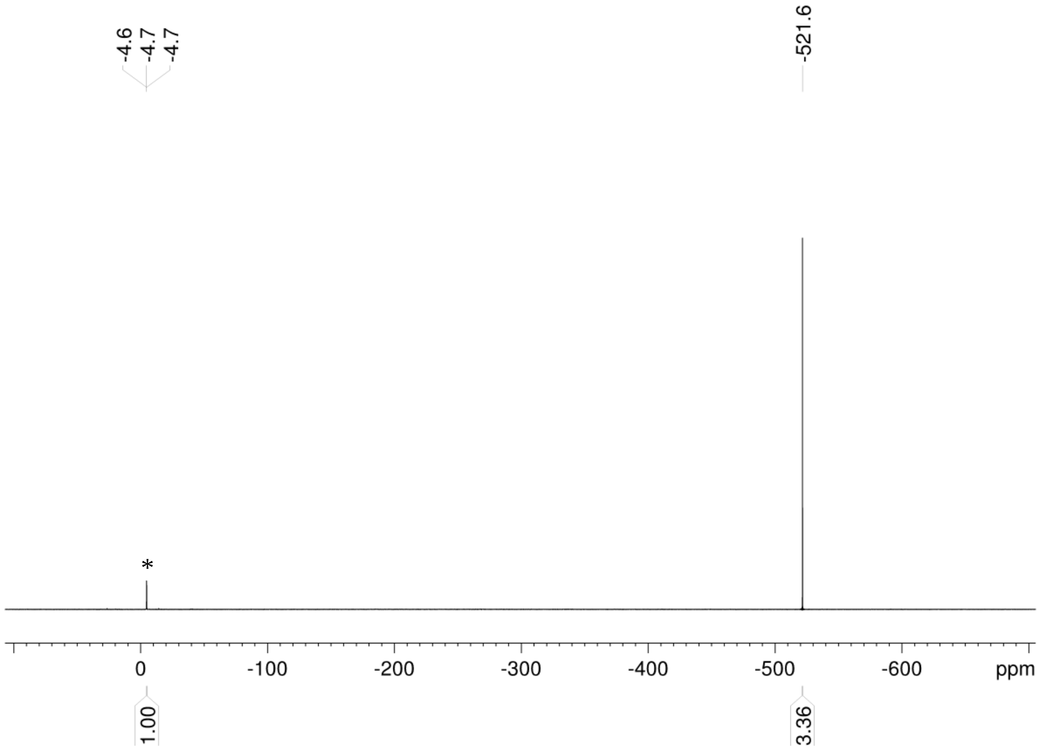


**Supplementary Figure 9.** $^{31}$P NMR spectrum after extraction of $P_4$@**C** with toluene. The NMR spectrum was recorded with a $C_6D_6$ capillary including $PPh_3$ (*) as internal standard.

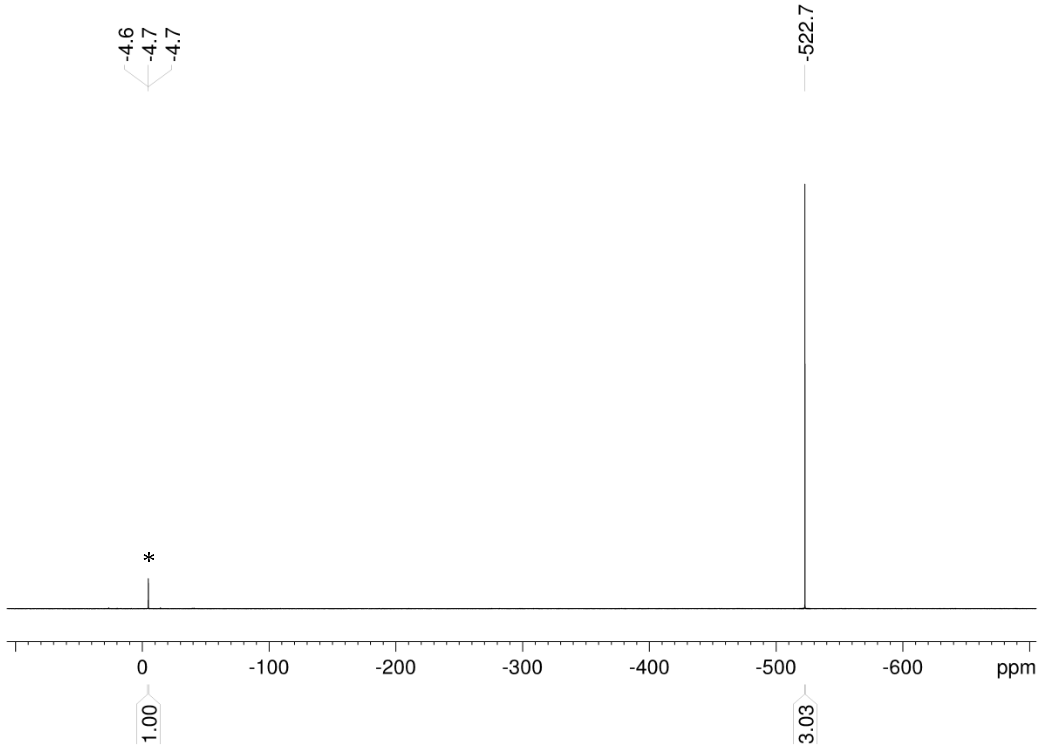


**Supplementary Figure 10.** $^{31}P$ NMR spectrum after extraction of $P_4$@**C** with dichloromethane. The NMR spectrum was recorded with a $C_6D_6$ capillary including $PPh_3$ (*) as internal standard.

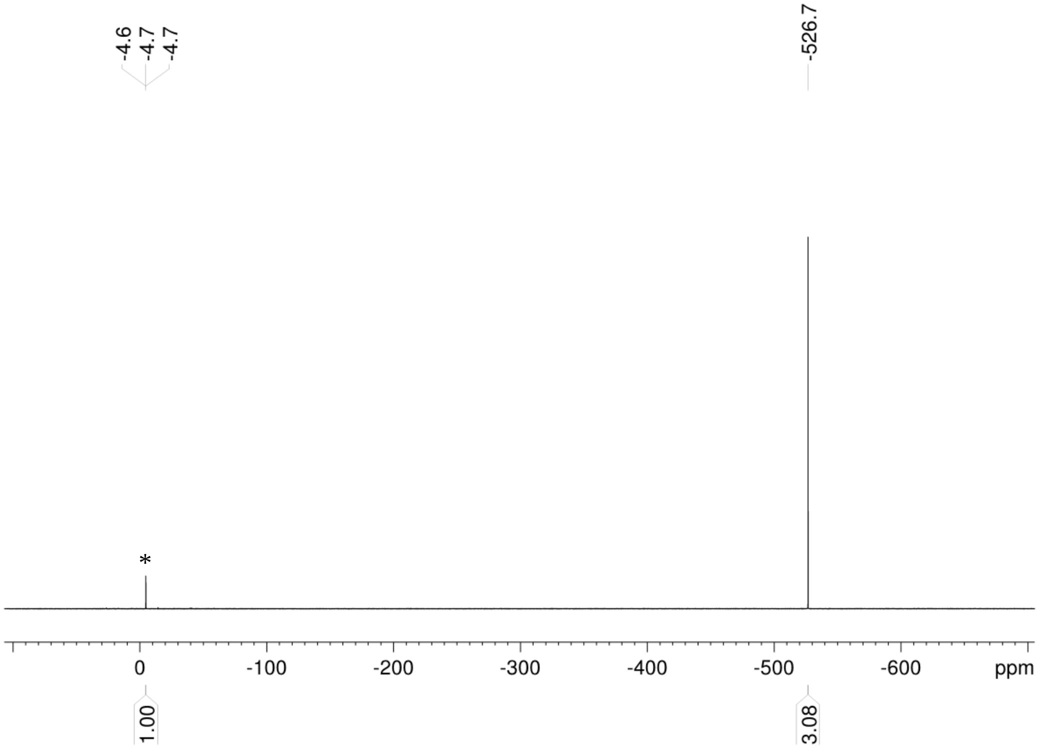


**Supplementary Figure 11.** $^{31}P$ NMR spectrum after extraction of $P_4$@**C** with thf. The NMR spectrum was recorded with a $C_6D_6$ capillary including $PPh_3$ (*) as internal standard.

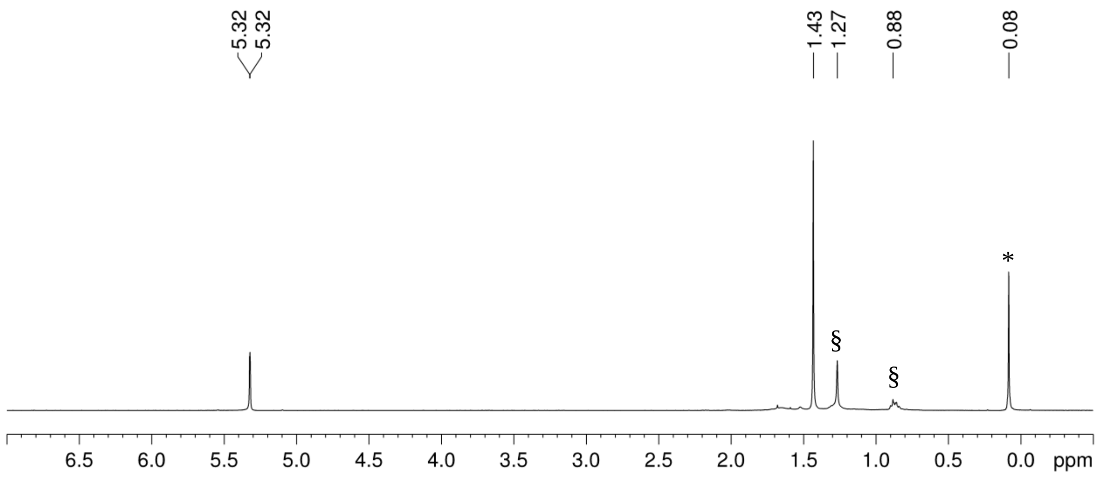


**Supplementary Figure 12.** $^1$H NMR spectrum of [Cp*Fe(η$^5$-$P_5$)] in $CD_2Cl_2$ at 300 K. Some impurities are due to residual solvents (§) and silicon grease (*).

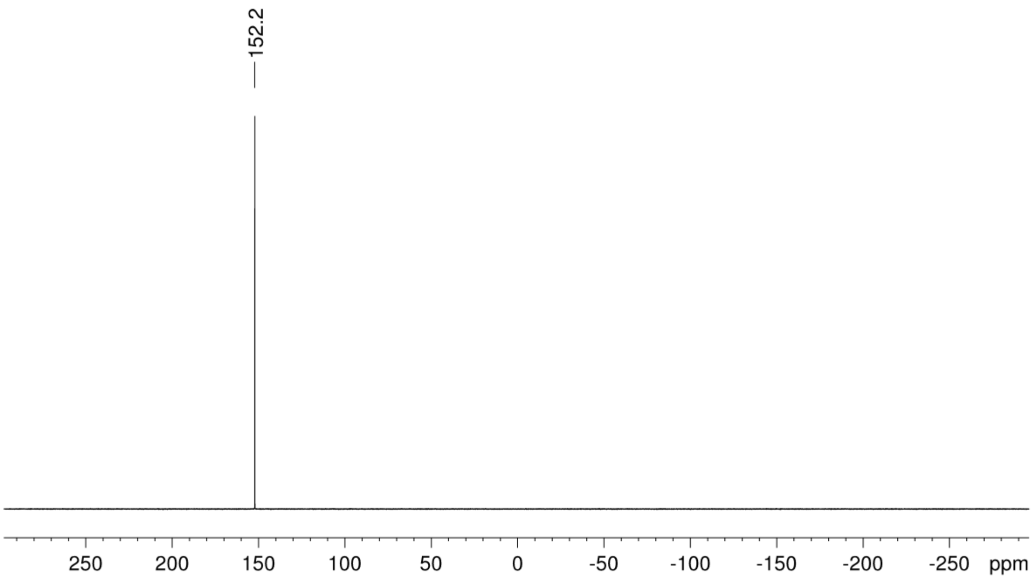


**Supplementary Figure 13.** $^{31}P$ NMR spectrum of [Cp*Fe($\eta^5$-$P_5$)] in $CD_2Cl_2$ at 300 K.

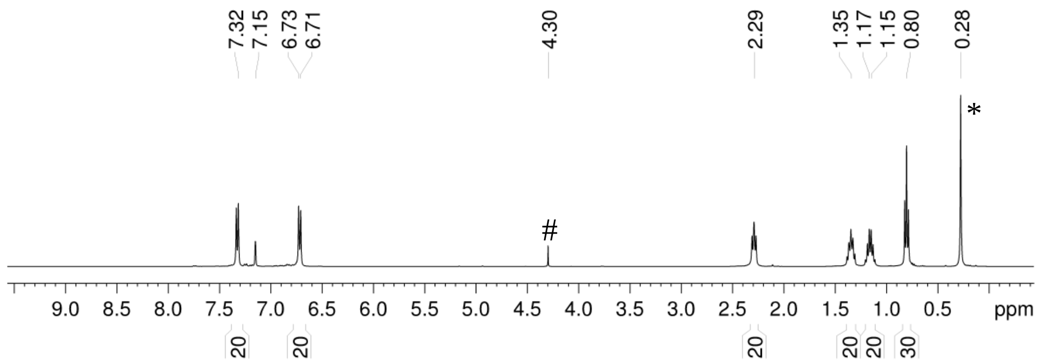


**Supplementary Figure 14.** $^1$H NMR spectrum of [{Cp$^{BIG}$Fe(CO)$_2$}$_2$(μ,η$^{1:1}$-P$_4$)] in $C_6D_6$ at 300 K. Some impurities due to silicon grease (*) and dichloromethane (#).

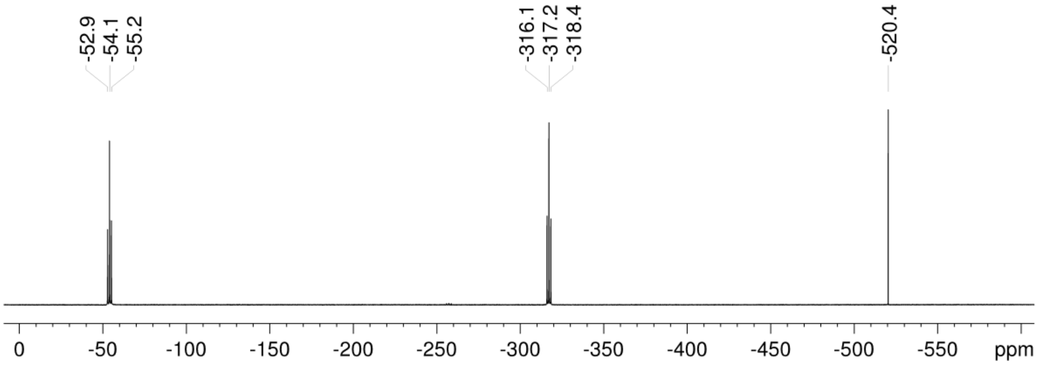


**Supplementary Figure 15.** $^{31}$P NMR spectrum of [{Cp$^{BIG}$Fe(CO)$_2$}$_2$(μ,η$^{1:1}$-P$_4$)] in $C_6D_6$ at 300 K. Some unreacted $P_4$ extracted with toluene (*δ* = -520.4 ppm) is still present.

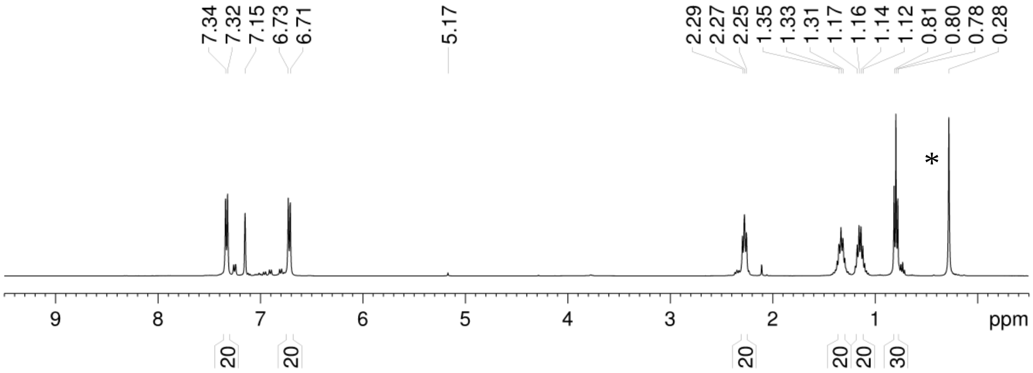


**Supplementary Figure 16.** $^{1}H$ NMR spectrum of [{$Cp^{BIG}Fe(CO)_2$}$_2$($\mu,\eta^{1:1}$-$As_4$)] in $C_6D_6$ at 300 K. Signal marked with * is due to silicon grease.

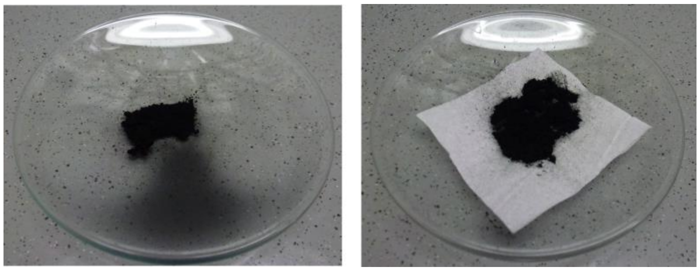

**Supplementary Figure 17.** Solid $P_4$@**C** on a glass on the lab bench (left) and on a tissue on a glass on the lab bench.

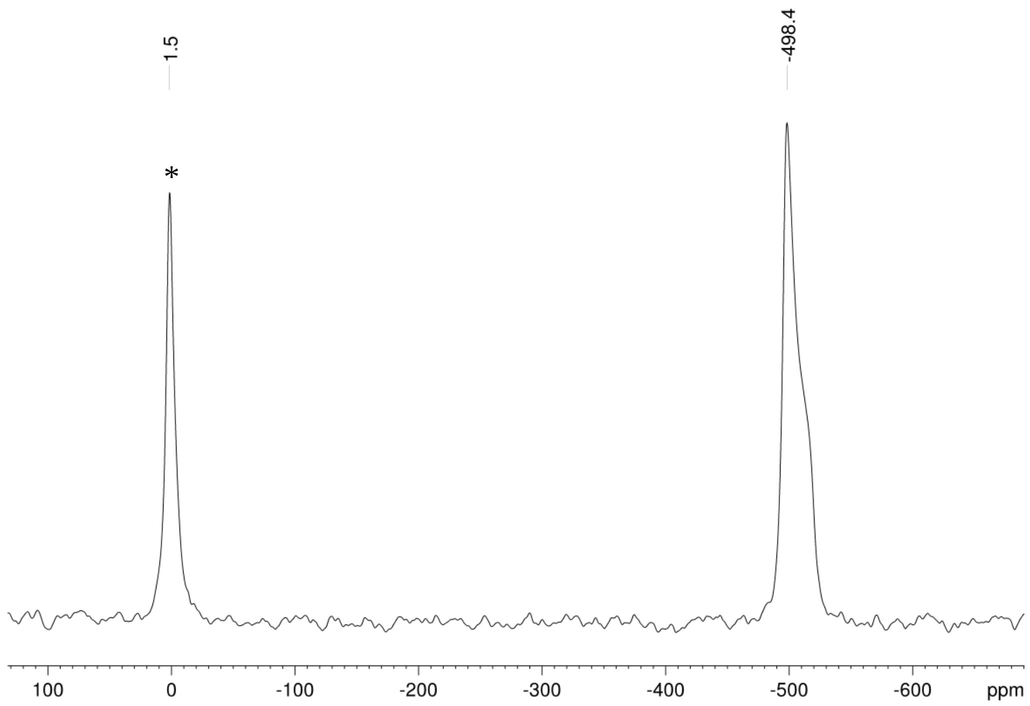


**Supplementary Figure 18.** $^{31}P\{^{1}H\}$ MAS NMR spectrum of $P_4$@**C** after storage for 3 weeks in air. Signal marked with * is due to oxidation and hydrolysis of traces of $P_4$ on the surface of the material, which can be removed by washing with MeOH.

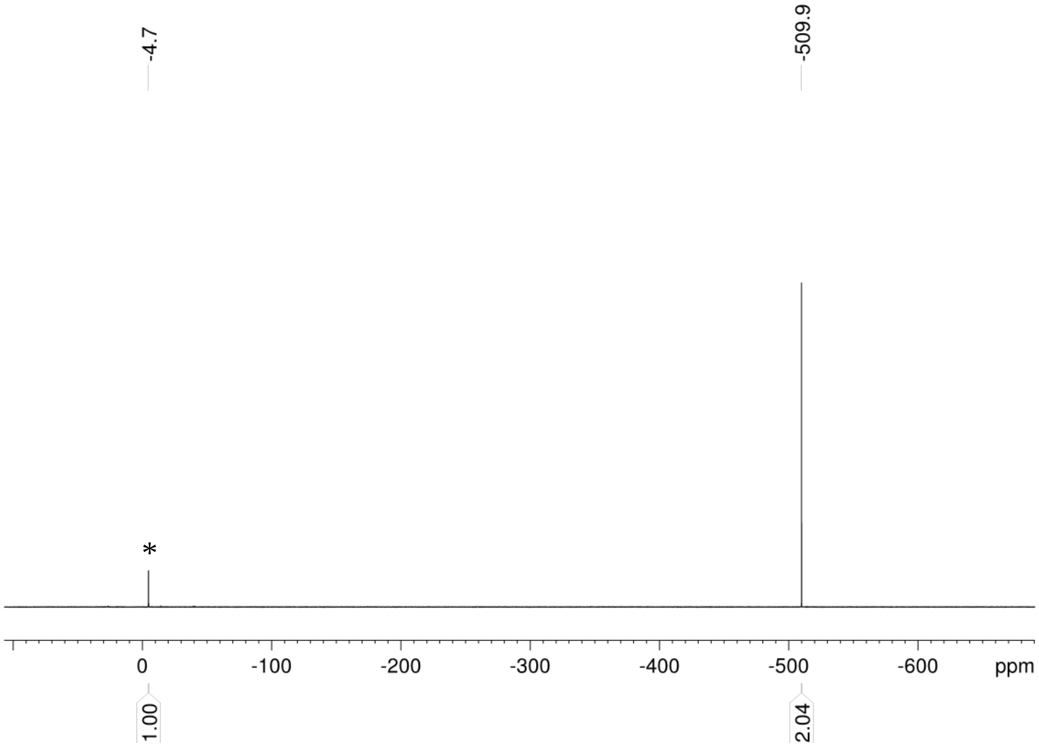


**Supplementary Figure 19.** $^{31}P$ NMR spectrum after extraction of $P_4$@**C** with $CS_2$. The NMR spectrum was recorded with a $C_6D_6$ capillary including $PPh_3$ (*) as internal standard.

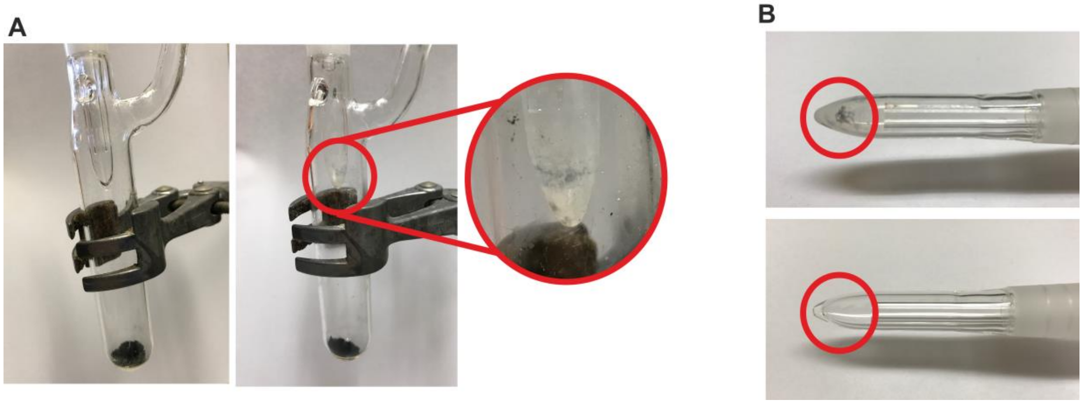


**Supplementary Figure 20.** (**A**) Pictures of the sublimation apparatus before (left) and after (right) sublimation of $P_4$@**C**. The red circle highlights the grey solid which covers the cold finger after sublimation. (**B**) Pictures of the sublimation finger after sublimation of $As_4$@**C** stored in air before (up) and after (down) treatment with concentrated $HNO_3$.

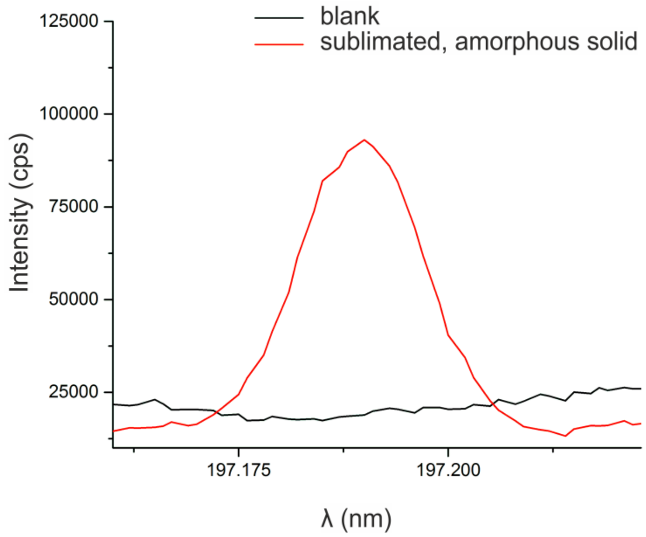


**Supplementary Figure 21.** Spectrum of the ICP-OES measurement of the blank (black) and the dissolved sublimated amorphous grey solid (red).

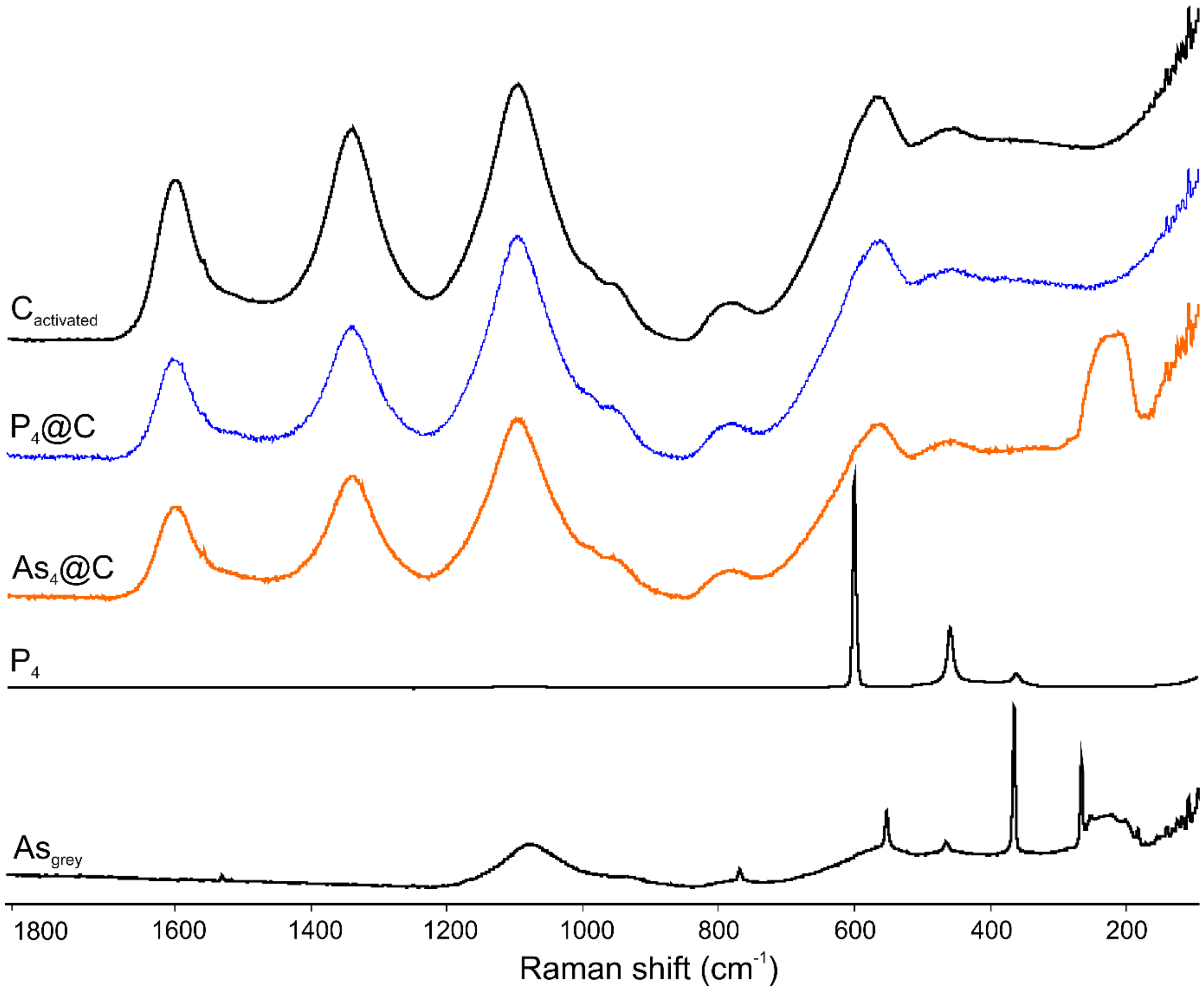


**Supplementary Figure 22.** Raman spectra (excitation wavelength 532 nm) of activated substrate (black), $P_4$@C (blue), $As_4$@C (orange), solid $P_4$ (black, middle) and grey As (black, bottom). For $As_4$@C the band at 220 nm is most likely attributable to traces of gray As on the surface of the material, generated by the high light sensitivity of yellow $As_4$ during the preparation of the material. The Raman spectra were recorded on a Thermo Fisher Scientific DXR-Smart-Raman spectrometer (Excitation laser 532 nm).

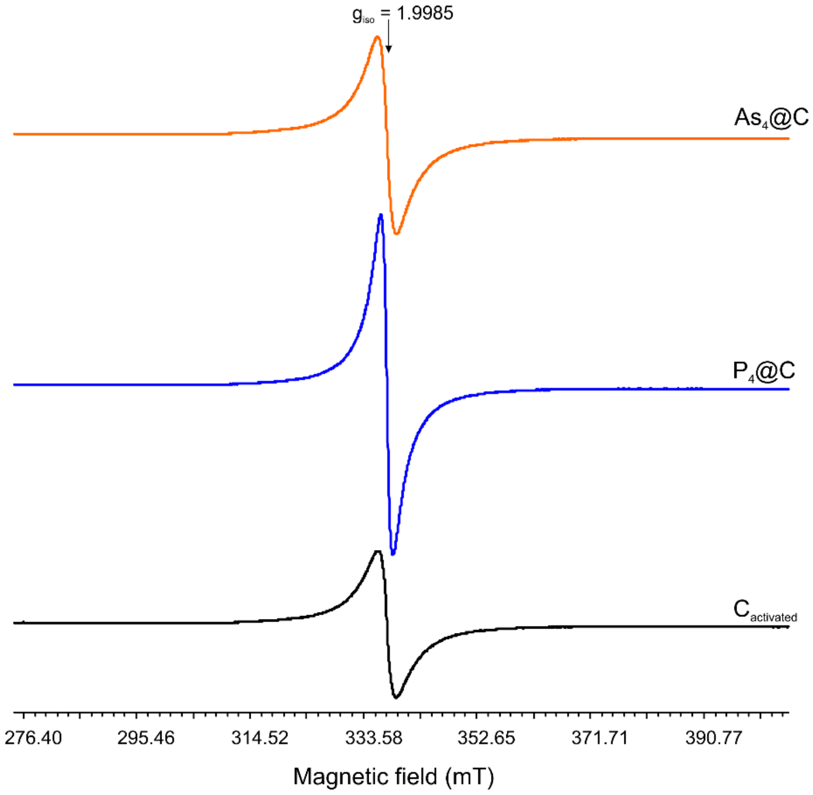


**Supplementary Figure 23.** X-Band EPR spectrum of activated C-substrate (black), $P_4$@C (blue) and $As_4$@C (orange). In all cases $g_{iso}$ = 1.9985. The X-Band EPS spectra were recorded on a Magnettech MiniScope MS400 spectrometer with 9 GHz microwave frequency.
